# Local enrichment of fatty acid-binding protein 4 in the pericardial cavity of cardiovascular disease patients

**DOI:** 10.1371/journal.pone.0206802

**Published:** 2018-11-05

**Authors:** Atlanta G. I. M. Elie, Maria Bloksgaard, Wai Y. Sun, Kangmin Yang, Andy W. C. Man, Aimin Xu, Akhmadjon Irmukhamedov, Lars P. Riber, Yu Wang, Jo G. R. De Mey

**Affiliations:** 1 Department of Cardiovascular and Renal Research, Institute of Molecular Medicine, University of Southern Denmark, Odense C, Denmark; 2 State Key Laboratory of Pharmaceutical Biotechnology, Li Ka Shing Faculty of Medicine, The University of Hong Kong, Hong Kong, China; 3 Department of Pharmacology and Pharmacy, Li Ka Shing Faculty of Medicine, The University of Hong Kong, Hong Kong, China; 4 Department of Medicine, The University of Hong Kong, Hong Kong, China; 5 Department of Cardiac, Thoracic and Vascular Surgery, Odense University Hospital, Odense C, Denmark; Institut d'Investigacions Biomediques de Barcelona, SPAIN

## Abstract

**Background:**

The pericardial fluid may be representative of the interstitium of the heart. The aim of this study was to discriminate in cardiovascular disease patients between adipocytokines that are produced locally by the heart and those supplied by the circulation.

**Methods:**

Enzyme-linked immunosorbent assays (ELISA) were used to determine levels of N-terminal pro-brain natriuretic peptide (NT-pBNP), fatty acid-binding protein 4 (FABP4), leptin, lipocalin-2, neutrophil elastase, proteinase-3, high sensitivity C-reactive protein (hsCRP) and adiponectin in venous plasma and pericardial fluid harvested during elective cardio-thoracic surgery (n = 132–152).

**Results:**

In pericardial fluid compared to plasma, the levels were significantly smaller (p < 0.001) for leptin, lipocalin-2, neutrophil elastase, proteinase-3, hsCRP and adiponectin. For these biomarkers, the ratio of pericardial fluid-to-plasma level ([PF]/[P], median (interquartile range)) was 0.65 (0.47–1.01), 0.78 (0.56–1.09), 0.23 (0.11–0.60), 0.17 (0.09–0.36), 0.14 (0.08–0.35), and 0.25 (0.15–0.34), respectively. In contrast, pericardial fluid was significantly enriched (p < 0.001) in NT-pBNP ([PF]/[P]: 1.9 (1.06–2.73)) and even more so for FABP4 ([PF]/[P]: 3.90 (1.47–9.77)). Moreover, in pericardial fluid, the adipocytokines interrelated all significantly positive and correlated negative to hsCRP, whereas for NT-pBNP only a significantly positive correlation with adiponectin was found. These interrelations were distinct from those in the plasma, as were the correlations of the pericardial biomarkers with patient characteristics compared to plasma.

**Conclusions:**

In cardiovascular disease patients, the pericardial cavity is a distinct adipocytokine microenvironment in which especially FABP4 is mainly derived from the heart.

## Introduction

Adipocytokines are a link between obesity, inflammation and cardiovascular diseases. In obesity, adipose tissue can become dysfunctional, resulting in a change in cellular composition towards a pro-inflammatory phenotype[1]. Moreover, obesity is characterized by systemic low-grade inflammation reflected by elevated circulating levels of high sensitivity C-reactive protein[2]. Most adipocytokines identified in adipose depots are pro-inflammatory and upregulated in hypertrophic adipose tissue[3] and can via the circulation promote metabolic and cardiovascular diseases[1]. For instance, patients with coronary artery disease have increased expression of pro-inflammatory adipocytokines in epicardial adipose tissue compared to non-coronary artery disease patients, where expression of the anti-inflammatory adiponectin is decreased[4]. We recently determined the levels of fatty acid-binding protein 4 (FABP4), leptin, and adiponectin in not only the circulation but also the pericardial fluid from a small number of cardiovascular disease patients[5]. This fluid can be representative for the interstitium of the heart. Pericardial fluid contains many bioactive compounds, such as natriuretic peptides, endothelin-1[6] and angiotensin II[7]. The local concentrations of these signaling molecules are particularly elevated in cardiovascular disease patients compared to the corresponding plasma levels and can have direct effects on cardiac function. Current knowledge of the composition and potential pathophysiological or diagnostic significance of the pericardial fluid is, however, incomplete.

The aim of this study was to discriminate, in cardiovascular disease patients, between locally produced adipocytokines, related to adipose tissue inflammation and myocardial dysfunction, measured in the pericardial fluid and those supplied by the circulation. We tested the hypothesis that the pericardial cavity of cardiovascular disease patients is a unique adipocytokine microenvironment, the composition of which can only partly be predicted from the circulation. For this, levels of FABP4, leptin, lipocalin-2, neutrophil elastase, proteinase-3, and adiponectin were measured and compared in plasma and pericardial fluid, harvested from a considerably larger group of patients (compared to previous studies[5, 8–11]) undergoing elective cardiothoracic surgery. N-terminal pro-brain natriuretic peptide (NT-pBNP) and hsCRP were included as markers of left ventricular dysfunction[12] and systemic inflammation[13], respectively. In this cross-sectional observational study, we deliberately included a broad variety of cardiovascular disease patients to obtain wide ranges of local and circulating adipocytokine concentrations. In contrast to most previous analyses of human pericardial fluid, we compared in the same individuals, concentrations in pericardial fluid to those in the venous plasma in order to discriminate between endocrine and local phenomena. Compared to our previous study[5], the novelty of the present work includes: i) a four times larger study population, ii) measurements of eight (instead of three) biomarkers, iii) attention for not only adipocytokines but also indices of left ventricular dysfunction and inflammation and iv) analyses of the cross-correlations between these biomarkers in the circulation and in the local pericardial microenvironment.

## Material and methods

### Subjects, sampling and ethics

All experiments were performed in accordance with institutional guidelines and were approved by The Regional Committees on Health Research Ethics for Southern Denmark (S-20100044 and S-20140202). Investigations were performed conform the principles outlined in the Declaration of Helsinki and informed written consent was obtained from all patients prior to collection of the biopsies and information from their medical records. The day before elective coronary artery bypass grafting (CABG) and/or cardiac valve replacement (VR) surgery, peripheral venous blood (5 mL) was drawn into pyrogen-free tubes containing K_2_EDTA and aprotinin. Pericardial fluid samples (2–5 mL), also collected in K_2_EDTA and aprotinin, were obtained right after median sternotomy and section of the pericardium. All samples were centrifuged (3220 G for ten minutes at 4°C) and the supernatants were stored at -80°C until analysis. The patients included in this study were free of pericarditis, pericardial effusion and pericardial tamponade and only pericardial fluid samples without visible blood contaminants were considered for analysis. Routine plasma analyses for HbA1c, lipids and creatinine were performed at the Odense University Hospital Clinical Biochemistry laboratory. Clinical information was obtained from the patients’ medical records and patient characteristics were disclosed to the researchers after finalization of their data collection. These and general patient characteristics are summarized in Table 1. The study addressed samples of a total of 159 patients that included the 37 patients from which a small number of characteristics and biomarker concentrations were previously reported [5].

**Table 1:** Patient characteristics and biomarker concentrations.

**Variable**	**Value**	**Missing information (%)**
N	159	
CABG / VR / both (%)#	53 / 34 / 13	0
Age (years)	69 (62–74)	0
Male (%)	81	0
Smoking Y / F / N (%)	21 / 49 / 27	3
BMI (kg/m^2^)	28 ± 4.4	0.6
Systolic BP (mmHg)	137 ± 19	1.3
Diastolic BP (mmHg)	74 ± 13	0.6
Ejection fraction (%)	55 (50–60)	11.3
Known hypertension (%)	64	9.4
Type 2 diabetes (%)	25	6.3
Dyslipidemia (%)	84	1.9
**Circulating Diagnostics**	**Value**	**Missing information (%)**
HbA1c (mmol/mol)	39 (35–43)	7.5
Total cholesterol (mmol/L)	3.9 (3.3–4.6)	7.5
LDL cholesterol (mmol/L)	2.1 (1.7–2.7)	8.2
HDL cholesterol (mmol/L)	1.1 (0.9–1.3)	7.5
Triglycerides (mmol/L)	1.3 (1.0–1.9)	8.2
Creatinine (μmol/L)	89 (75–101)	2.5
**Pericardial fluid concentrations**	**Value**	**Missing information (%)**
NT-pBNP (μg/L)	0.48 (0.41–0.85)	4.4
FABP4 (μg/L)	80 (42–194)	5.0
Leptin (μg/L)	11 (5–19)	4.4
Lipocalin-2 (μg/L)	31 (24–43	4.4
Neutrophil elastase (μg/L)	19 (8.8–45)	5.7
Proteinase-3 (μg/L)	8.8 (4.3–17)	5.0
hsCRP (mg/L)	0.32 (0.29–0.45)	4.4
Adiponectin (mg/L)	3.7 (2.4–5.7)	4.4
Total protein (g/L)	25 (21–32)	4.4
**Plasma Concentrations**	**Value**	**Missing information (%)**
NT-pBNP (μg/L)	0.25 (0.21–0.43)	11.3
FABP4 (μg/L)	20 (10–34)	11.9
Leptin (μg/L)	17 (8.3–29)	11.3
Lipocalin-2 (μg/L)	37 (29–50)	11.3
Neutrophil elastase (μg/L)	66 (53–88)	11.3
Proteinase-3 (μg/L)	46 (34–65)	11.3
hsCRP (mg/L)	3,0 (1,4–4,5)	11.3
Adiponectin (mg/L)	15 (11–23)	11.3
Total protein (g/L)	67 (59–76)	11.3
**Medications**	**Value**	**Missing information (%)**
ACEI / ARB (%)	29 / 17	0
Aspirin (%)	63	0
β-blocker (%)	57	0
Biguanide (%)	19	0
Ca^2+^-antagonist (%)	36	0
Diuretic (%)	34	0
Statin (%)	70	0

Categorical data are shown as % of the study group, normally distributed continuous variables as mean ± SD, non-normally distributed continuous variables as median value (interquartile range). #, type of surgery; CABG, coronary artery bypass surgery; VR, valve replacement; Y; yes. F; former. N; never; BMI, body mass index; BP, blood pressure; NT-pBNP, N-terminal pro-brain natriuretic peptide; FABP4, fatty acid-binding protein 4; hsCRP, high sensitivity C-reactive protein; ACEI/ARB, inhibitor of angiotensin converting enzyme or antagonist of angiotensin AT_1_ receptors.

### Measurements of biomarker concentration

Plasma and pericardial fluid levels of NT-pBNP, FABP4, lipocalin-2, neutrophil elastase, proteinase-3, adiponectin and hsCRP were measured using in-house ELISA kits and a hsCRP assay, respectively (http://www.pharma.hku.hk/sweb/antibody/ELISA.php), as described[14, 15]. Plasma and pericardial fluid levels of leptin were determined with an ELISA kit from Biovendor Laboratories (Brno, Czech Republic) as described[15]. After 100 times dilution, the total protein in plasma and pericardial fluid was assayed using Pierce BCA Protein Assay Kit (# 23225, Thermo Fisher Scientific, Massachusetts, United States) according to the instructions of the manufacturer. All samples were analysed in duplicate.

### Statistics

Continuous variables that were normally distributed (Kolmogorov-Smirnov test) are shown as mean ± standard deviation (SD). Concentrations of biomarkers and continuous variables that were not normally distributed are shown as median value and interquartile range. Paired comparisons between observations in plasma and pericardial fluid of the same individuals were performed with Wilcoxon matched-pairs signed rank tests. Associations between the levels of biomarkers in plasma and pericardial fluid and between biomarker concentrations and continuous clinical variables were assessed with Spearman correlation tests. Evaluation between all categorical clinical variables and biomarker levels were performed using Mann-Whitney U tests. Statistical analyses were performed with GraphPad Prism v6.05 (GraphPad Software Inc, San Diego, Ca, USA). Differences and associations were considered statistically significant at *p* < 0.05. In the tables and figures, the biomarkers are ranked low to high according to the molecular weight of their most abundant soluble oligomer reported in the literature[16, 17].

## Results

### Patient characteristics

Table 1 summarizes characteristics of the study population. The patients underwent coronary artery bypass (53%), cardiac valve replacement (34%) or both (13%), indicating that 66% suffered from coronary artery disease. Most patients were elderly (70 years), overweight (BMI >27), males (81%) previously diagnosed with dyslipidemia (84%), prescribed statins (70%) and had normal plasma lipid levels. Most had a history of smoking (70% former or current smoker). Sixty-four percent were hypertensive (defined as mean arterial pressure >105 mmHg or preoperative antihypertensive pharmacotherapy) and a small fraction had type 2 diabetes (25%) and was prescribed a biguanide (19%). On average, the study population had a plasma HbA1c level within normal range (HbA1_c_ < 42 mmol/mol). The creatinine values indicated normal kidney function. Most patients had been prescribed a combination of low-dose aspirin, inhibitors of the renin angiotensin system and diuretics.

### Pericardial fluid levels of biomarkers

**Fig 1:**
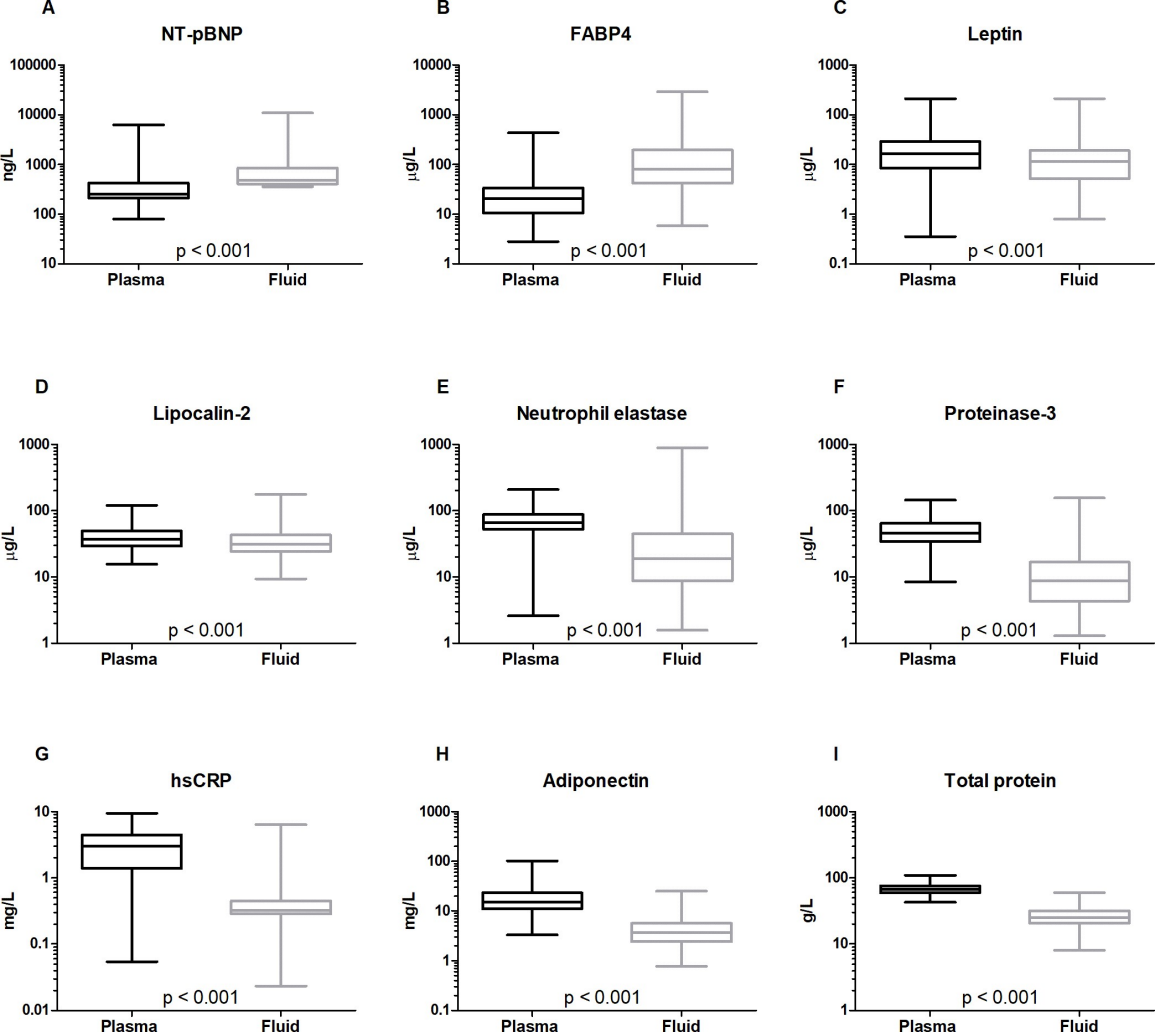
**Venous plasma and pericardial fluid concentrations of NT-pBNP (A), FABP4 (B), leptin (C), lipocalin-2 (D), neutrophil elastase (E), proteinase-3 (F), hsCRP (G), adiponectin (H) and total protein (I) in cardiac and vascular disease patients (n = 132–152).** The box plots illustrate median values, interquartile range and minimal and maximal concentrations compared by Wilcoxon matched-pairs signed rank test. NT-pBNP, N-terminal pro-brain natriuretic peptide; FABP4, fatty acid-binding protein 4; hsCRP, high sensitivity C-reactive protein. The proteins were ordered (low to high) according to the molecular weight of their reported most abundant soluble oligomer.

**Fig 2:**
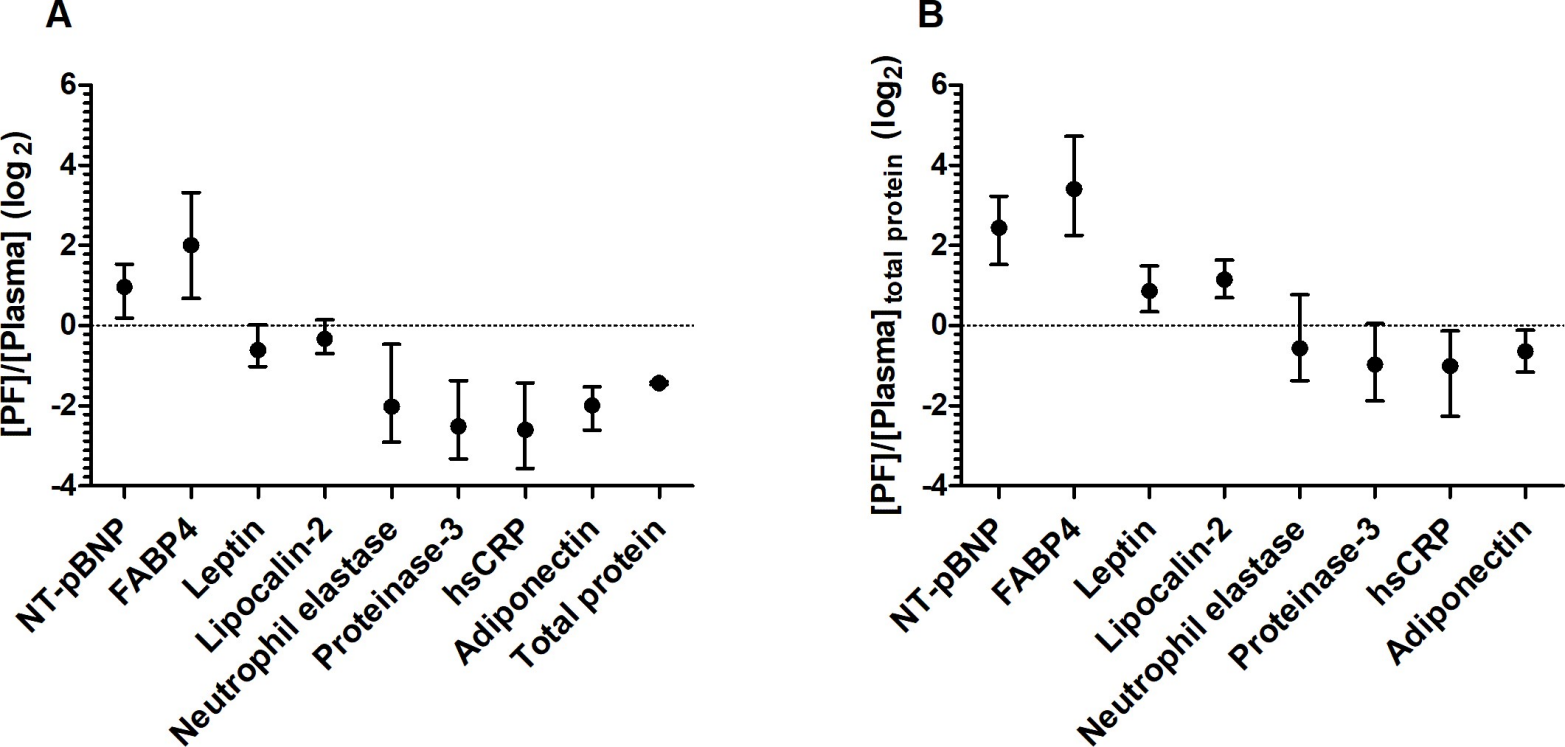
**A) Ratios of pericardial fluid-to-plasma level ([PF]/[Plasma], n = 132–152) of putative biomarkers and total protein, and B) ratios of the relative abundance of these biomarkers in the pericardial fluid and plasma ([PF]/[Plasma]**_**total protein**_**, n = 134–136).** Shown are the log_2-_transformed median (interquartile range) ratios. NT-pBNP, N-terminal pro-brain natriuretic peptide; FABP4, fatty acid-binding protein 4; hsCRP, high sensitivity C-reactive protein. The proteins were ordered (low to high) according to the molecular weight of their reported most abundant soluble oligomer.

In pericardial fluid, the concentrations of all 8 biomarkers and the total protein concentration differed significantly from those in the plasma (Fig 1, p < 0.001). Compared to plasma, they were higher for NT-pBNP and FABP4 but lower for leptin, lipocalin-2, neutrophil elastase, proteinase-3, hsCRP, adiponectin and total protein. According to calculated pericardial fluid-to-plasma ratio’s ([PF]/ [P]), median (interquartile range), Fig 2A), NT-pBNP was two times more abundant in the local microenvironment around the heart than in the circulation ([PF]/ [P], 1.9 (1.06–2.73)) and FABP4 was on average four times enriched in pericardial fluid compared to plasma (3.90 (1.47–9.77)). For leptin and lipocalin-2, concentrations were lower in pericardial fluid than plasma ([PF]/ [P]: 0.65 (0.47–1.01) and 0.78 (0.56–1.09), respectively). For the other biomarkers, pericardial fluid concentrations were markedly lower compared to plasma levels ([PF]/ [P]: neutrophil elastase, 0.23 (0.11–0.60); proteinase-3, 0.17 (0.09–0.36); hsCRP, 0.14 (0.08–0.35); adiponectin, 0.25 (0.15–0.34). The total protein concentration in pericardial fluid was 0.37 (0.29–0.47) times that observed in the plasma of the same patients. When calculating these biomarker concentrations as a fraction of the total protein concentration of each compartment ([PF]/ [P]_total protein_, median (interquartile range), Fig 2B), NT-pBNP was more than 5 times and FABP4 even more than 10 times enriched ([PF]/[P]_total protein_ (5.4 (2.9–9.4) and (10.6 (4.7–26), respectively). Now, leptin (1.8 (1.3–2.8)) and lipocalin-2 (2.2 (1.6–3.1) also seemed significantly enriched in the pericardial cavity. Neutrophil elastase (0.68 (0.39–1.7)), proteinase-3 (0.51 (0.27–1.04)), hsCRP (0.50 (0.21–0.91)), and adiponectin (0.64 (0.45–0.92)), on the other hand, made up a significantly smaller fraction of the total protein concentration in pericardial fluid compared to plasma.

### Relations between levels of biomarkers

**Fig 3:**
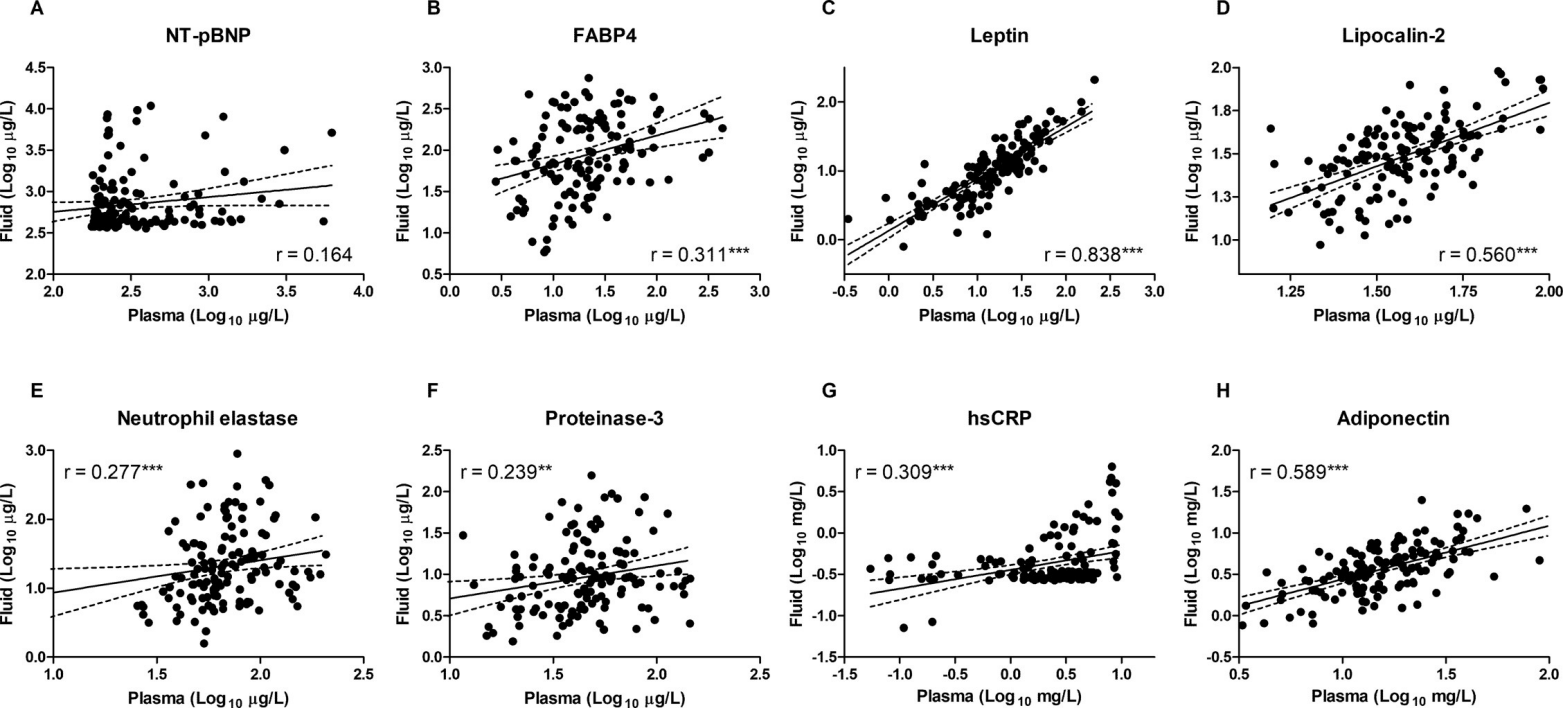
**Associations between venous plasma and pericardial fluid concentrations of NT-pBNP (A), FABP4 (B), leptin (C), lipocalin-2 (D), neutrophil elastase (E), proteinase-3 (F), hsCRP (G), and adiponectin (H) in cardiac and vascular disease patients (n = 132–152).** The double logarithmic plots by Spearman correlation illustrate the relationship between pericardial fluid concentrations and circulating levels of the biomarkers. NT-pBNP, N-terminal pro-brain natriuretic peptide; FABP4, fatty acid-binding protein 4; hsCRP, high sensitivity C-reactive protein; ***, P < 0.001. The proteins were ordered (low to high) according to the molecular weight of their reported most abundant soluble oligomer.

Regardless of the different concentrations in the two compartments, the pericardial fluid concentrations correlated positively with the plasma concentrations for each biomarker investigated except NT-pBNP (Fig 3).

**Fig 4:**
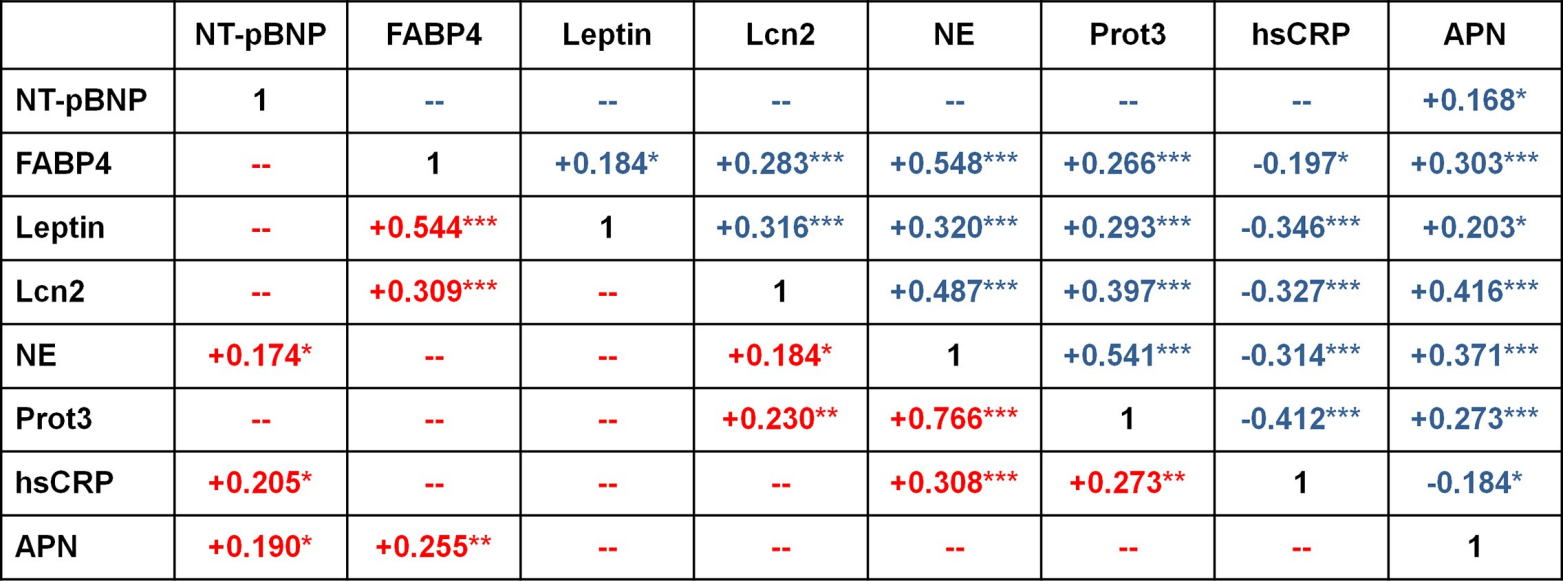
Relationships between the concentrations of biomarkers in pericardial fluid (in blue) and venous plasma (in red) of cardiovascular disease patients. Statistically significant correlations between continuous variables are indicated by Spearman correlation coefficient and *, p < 0.05; **, p < 0.01 and ***, p < 0.001. Peptides and proteins are ordered (left to right and top to bottom) according to the molecular weight of their reported most abundant soluble oligomer. N = 139–152. NT-pBNP, N-terminal pro-brain natriuretic peptide; FABP4, fatty acid-binding protein 4; Lcn2, lipocalin-2; NE, neutrophil elastase; Prot3, proteinase-3; hsCRP, high sensitivity C-reactive protein; APN, adiponectin; —, not statistically significant.

In pericardial fluid, the levels of FABP4, leptin, lipocalin-2, neutrophil elastase, proteinase-3 and adiponectin were positively interrelated and each displayed an inverse correlation to the local concentrations of hsCRP (Fig 4). In the plasma, no inverse relationships and fewer statistically significant positive relationships were observed (Fig 4). The relationships that were found in both compartments were those between NT-pBNP and adiponectin, between FABP4 and adiponectin, between FABP4 and leptin, between FABP4 and lipocalin-2, between lipocalin-2 and neutrophil elastase, between neutrophil elastase and proteinase-3, and between lipocalin-2 and proteinase-3 (Fig 4).

### Relations to patient properties

In the pericardial cavity, NT-pBNP levels were significantly positively correlated to age and cardiac valve disease. FABP4 levels correlated positively with plasma creatinine concentrations. Leptin related positively to BMI and circulating triglycerides. Lipocalin-2 correlated positively to age and plasma creatinine. Neutrophil elastase was positively linked to cardiac valve disease. Adiponectin correlated negatively with ischemic heart disease, type 2 diabetes, plasma triglycerides, and use of a biguanide, but positively with age, and circulating HDL cholesterol (Table 2).

**Table 2:** Associations of pericardial fluid levels of biomarkers with patient properties.

**Patient property**	**NT-pBNP**	**FABP4**	**Leptin**	**Lcn2**	**NE**	**Prot3**	**hsCRP**	**APN**
Surgery type	+VR#*	ns	ns	ns	+VR#*	ns	ns	+VR#***
Age	+0.182*	ns	ns	+0.300***	ns	ns	ns	+0.409***
BMI	ns	ns	+0.396***	ns	ns	ns	ns	ns
Type 2 diabetes	ns	ns	ns	ns	ns	ns	ns	–**
EF ≤ 50%	ns	ns	ns	ns	ns	ns	ns	ns
**Plasma**	**NT-pBNP**	**FABP4**	**Leptin**	**Lcn2**	**NE**	**Prot3**	**hsCRP**	**APN**
HbA1c	ns	ns	ns	ns	ns	ns	ns	ns
HDL cholesterol	ns	ns	ns	ns	ns	ns	ns	+0.298***
Triglycerides	ns	ns	+0.214*	ns	ns	ns	ns	–0.199*
Creatinine	ns	+0.212**	ns	+0.310***	ns	ns	ns	ns
**Medications**	**NT-pBNP**	**FABP4**	**Leptin**	**Lcn2**	**NE**	**Prot3**	**hsCRP**	**APN**
Statin	ns	ns	ns	ns	ns	ns	ns	ns
ACE/ARB	ns	ns	ns	ns	ns	ns	ns	ns
Biguanide	ns	ns	ns	ns	ns	ns	ns	–*

Statistically significant correlations to categorical or continuous variables are indicated as follows:

*, p < 0.05

**, p < 0.01 and

***, p < 0.001.

For continuous variables, the Spearman correlation coefficient is shown as well. NT-pBNP, N-terminal pro-brain natriuretic peptide; FABP4, fatty acid-binding protein 4; Lcn2, lipocalin-2; NE, neutrophil elastase; Prot3, proteinase-3; hsCRP, high sensitivity C-reactive protein; APN, adiponectin; ns, not statistically significant; (+) and (-), positive and negative correlation; EF, ejection fraction; #, valve replacement (VR) surgery compared to coronary artery bypass grafting (CABG); other abbreviations as in Table 1.

In plasma, NT-pBNP concentrations were inversely correlated with being diabetic, use of statins and a biguanide. FABP4 was positively linked to cardiac valve disease, age and HDL cholesterol. Leptin correlated positively with BMI and triglycerides. The concentration of lipocalin-2 was positively related to age, creatinine and the use of inhibitors of the renin angiotensin system. Neutrophil elastase was negatively linked to HDL cholesterol and biguanide use. Proteinase-3 was also negatively linked to plasma HDL cholesterol levels, but positively linked to circulating creatinine. hsCRP was negatively correlated to HDL cholesterol levels whereas adiponectin was negatively correlated to BMI, type 2 diabetes, HbA1c concentrations, triglycerides and use of statins, but positively to cardiac valve disease, age and HDL cholesterol (Table 3).

**Table 3:** Associations of plasma levels of biomarkers with patient properties.

**Patient property**	**NT-pBNP**	**FABP4**	**Leptin**	**Lcn2**	**NE**	**Prot3**	**hsCRP**	**APN**
Surgery type	ns	+VR#**	ns	ns	ns	ns	ns	+VR#***
Age	ns	+0.192*	ns	+0.183*	ns	ns	ns	+0.346***
BMI	ns	ns	+0.312***	ns	ns	ns	ns	–0.344***
Type 2 diabetes	–**	ns	ns	ns	ns	ns	ns	–***
EF ≤ 50%	ns	ns	ns	ns	ns	ns	ns	ns
**Plasma**	**NT-pBNP**	**FABP4**	**Leptin**	**Lcn2**	**NE**	**Prot3**	**hsCRP**	**APN**
HbA1c	ns	ns	ns	ns	ns	ns	ns	–0.275***
HDL cholesterol	ns	+0.188*	ns	ns	–0.287***	–0.234**	–0.329***	+0.479***
Triglycerides	ns	ns	+0.233**	ns	ns	ns	ns	–0.399***
Creatinine	ns	ns	ns	+0.508***	ns	+0.201*	ns	ns
**Medications**	**NT-pBNP**	**FABP4**	**Leptin**	**Lcn2**	**NE**	**Prot3**	**hsCRP**	**APN**
Statin	–*	ns	ns	ns	ns	ns	ns	–***
ACE/ARB	ns	ns	ns	+*	ns	ns	ns	ns
Biguanide	–*	ns	ns	ns	–**	ns	ns	–***

Statistically significant correlations to categorical or continuous variables are indicated as follows

*, p < 0.05

**, p < 0.01 and

***, p < 0.001.

For continuous variables, the Spearman correlation coefficient is shown as well. Peptides and proteins are ordered (left to right and top to bottom) according to the molecular weight of their reported most abundant soluble oligomer. NT-pBNP, N-terminal pro-brain natriuretic peptide; FABP4, fatty acid-binding protein 4; Lcn2, lipocalin-2; NE, neutrophil elastase; Prot3, proteinase-3; hsCRP, high sensitivity C-reactive protein; APN, adiponectin; ns, not statistically significant; + and–, positive and negative correlation; EF, ejection fraction; #, valve replacement (VR) surgery compared to coronary artery bypass grafting (CABG); other abbreviations as in Table 1.

## Discussion

The main findings of this study are that i) in the pericardial cavity, the concentrations of all 8 biomarkers significantly differed from those in the plasma with NT-pBNP and FABP4 being more abundant and the remaining biomarkers less abundant, ii) the pericardial fluid concentrations of all biomarkers, except NT-pBNP, correlated positively with their plasma levels, iii) the pericardial fluid levels of FABP4, leptin, lipocalin-2, neutrophil elastase, proteinase-3 and adiponectin were strongly interrelated and negatively related to the pericardial fluid concentration of hsCRP, whereas in the venous plasma less and only positive interrelationships were observed, and that iv) pericardial fluid biomarker concentrations correlated with many patient characteristics for which no correlations were found in the plasma. These findings demonstrate that the pericardial cavity is a distinct adipocytokine microenvironment and indicate that especially FABP4 is produced by the heart in CVD patients.

Dysfunctional adipose tissue, such as hypertrophic adipose tissue, is associated with increased release of pro-inflammatory adipocytokines from adipocytes and immune cells[1, 3]. These adipocytokines are recognized as a link between inflammation and cardiovascular diseases[1]. Elevated circulating hsCRP and pro-inflammatory adipocytokines like FABP4, leptin and lipocalin-2 increase the risk for cardiovascular disease[1, 13, 18] by not only endocrine but also paracrine mechanisms[1]. Especially epicardial adipose tissue displays a pathogenic phenotype in cardiovascular disease patients[4]. In epicardial adipose tissue, expression of the anti-inflammatory adiponectin, in turn, is decreased in cardiovascular disease patients with hypertension[19], suffering from coronary artery disease[4], and in those displaying a combination of features of the metabolic syndrome[20].

The composition of the pericardial fluid may both reflect and contribute to the pathophysiology of the heart and its coronary vasculature.

We previously obtained suggestive evidence that the pericardial cavity in cardiovascular disease patients is a pro-inflammatory atherogenic micro-environment, distinct from the plasma[5]. In the present study, we studied a larger number of adipocytokines with a broad range of molecular weights from 15 kDa (FABP4) up to ≥ 250 kDa (adiponectin) in a larger number of patients. NT-pBNP was regarded as a control, exclusively produced by the myocardium[21] and hsCRP as a marker of systemic inflammation[13], mainly produced by the liver[22].

Pericardial fluid was sampled from patients with coronary artery or cardiac valve disease. During cardiothoracic surgery, opening of the pericardial cavity enables easy and safe sampling of pericardial fluid[5, 8]. Patient characteristics in this study are comparable to other studies reporting on pericardial fluid composition[5, 8–11]. Comparison of venous plasma from the day before surgery with the pericardial fluid at initiation of surgery is justified because previous reports have indicated that levels may only change after a couple of hours of surgery[23, 24].

Only a few studies compared pericardial fluid and plasma from patients suffering from heart failure or coronary artery disease[5, 8–11]. In the present study, the results demonstrate that the concentration of NT-pBNP, hsCRP and the adipocytokines differ between both compartments, where NT-pBNP and FABP4 were more abundant, and the other biomarkers less abundant in the pericardial fluid. Moreover, the interrelations of the protein biomarkers in the pericardial cavity were distinct from those in the plasma, as were the correlations of the pericardial biomarkers with patient characteristics compared to plasma. These data strengthen our conclusion that the pericardial cavity is a distinct micro-environment of the heart.

The pericardial fluid is regarded by some as an ultrafiltrate of the plasma[25], whereas others consider it as representative of the cardiac interstitium[26]. The pericardial fluid-to-plasma ratio, including the ratio as a fraction of the total protein concentration of the investigated proteins showed a strong relationship with molecular weight from a high ratio for small molecules (8.5 kDa for NT-pBNP, 15 kDa for FABP4, 16 kDa for leptin, and 25 kDa for lipocalin-2) to a lower ratio with increasing molecular weight (29 kDa for proteinase-3, 29.5 kDa for neutrophil elastase, 120 kDa for hsCRP, and > 250 kDa for adiponectin). The pericardial fluid-to-plasma ratio was larger than one for NT-pBNP. This was expected for a molecule that is predominantly produced by the myocardium[21]. It was also comparatively large for leptin and lipocalin-2 with ratios of 0.65 and 0.78 resp. which from the point of view of an ultrafiltrate (ratio of 0.25) cannot be regarded as such. These proteins can be produced by not only adipocytes but also cardiomyocytes and coronary smooth muscle cells especially in heart failure[27–30]. The largest pericardial fluid-to-plasma ratio was, however, observed for FABP4, which is in line with recent observations by Furuhashi et al. of FABP4 in human epicardial adipose tissue and coronary atherosclerotic plaques and of higher levels of FABP4 in the coronary sinus compared to the aortic root[31]. Moreover, the ratios as a fraction of the total protein were even higher, ranging between 1.8 for leptin and 10.6 for FABP4, strongly indicating cardiac production of also these adipocytokines. Conversely, the pericardial fluid-to-plasma ratio, including the ratio as a fraction of total protein was very low for adiponectin although it can be produced by epicardial adipose tissue[4, 19, 20] and cardiomyocytes[32]. It is not likely that this is due to the large molecular weight of adiponectin complicating its diffusion over the epicardial mesothelium. We previously reported a similar contribution of high molecular weight adiponectin to the total concentration of adiponectin in pericardial fluid and venous plasma[5]. Rather, the pro-inflammatory adipocytokine environment in the pericardial cavity/cardiac interstitium might suppress local production of adiponectin [4, 33, 34] and thereby limit cardiovascular protective effects.

hsCRP, an important risk factor and independent predictor of cardiovascular events[13] is mainly produced by the liver[22] but can also be expressed by human adipocytes[33] and immune cells[35]. In this study, circulating levels of hsCRP were positively related to those of the strongly interrelated neutrophil markers proteinase-3 and neutrophil elastase[36, 37] as reported before[38]. In the pericardial fluid, on the other hand, the concentration of hsCRP was inversely related to that of the still strongly interrelated proteinase-3 and neutrophil elastase. This suggests local proteolysis[39] and increased local permeability of hsCRP, by the neutrophil enzymes. The local concentration of other large molecular weight proteins like leptin and lipocalin-2, but not the small peptide fragment NT-pBNP, seems to be similarly affected (Fig 4).

In the systemic circulation of coronary artery disease[40] or heart failure patients[41], levels of NT-pBNP and of adiponectin are positively related independently from systemic inflammation. In our study of a broadly diverse group of patients, we confirmed this association in the venous plasma and observed it for the first time in the pericardial fluid. Natriuretic peptides have favorable effects on the distribution of adipose tissues in the body[42] and on the phenotype of adipocytes[43]. They stimulate the production of adiponectin by cultured human adipocytes[44] and increase the circulating levels of total and high molecular weight adiponectin when infused into the body[45]. Conversely, cardiomyocytes express low levels of adiponectin and its receptors[32] that are increased under experimental pathological conditions[46]. Also, adiponectin decreases cardiomyocyte hypertrophy and BNP mRNA expression[47] and reduces myocardial oxidative stress[48]. These and our present observations indicate both systemic and local interaction between a sign of myocardial dysfunction[21] and an adipocyte-derived cardiovascular protective and anti-inflammatory endocrine and paracrine mediator[18, 48]. Interplay between adipocytokines and inflammatory signals seem however to differ between locations as we observed strong interrelations of the levels of FABP4, lipocalin-2, proteinase-3 and neutrophil elastase in pericardial fluid/cardiac interstitium and to a much lesser extent in the circulation (Fig 4).

### Study limitations and perspectives

Our study is a cross-sectional observational study in very diverse patients with the aim to obtain broad ranges of local and circulating concentrations of adipocytokines and markers of heart failure and inflammation. For obvious ethical reasons, pericardial fluid could not be obtained from healthy volunteers. Our results may therefore be affected by for instance presence of comorbidities or the severity of chronic cardiovascular condition. The associations that we identified could be accidental and coincident rather than indicative of causal relationships. We determined pericardial fluid concentrations but could not consider the volume of the pericardial fluid. Echocardiography information on the pericardial space[10] was not available. Analyses of relations to patient demographic, diagnostic and pharmacotherapeutic characteristics were superficial at this stage.

Despite these limitations, our suggestive evidence of the pericardial space as a distinct adipocytokine micro-environment adds to the proposal that the pericardial space holds potential for improved diagnosis and local treatment[49]. It invites for more extensive proteomic analyses[50] that address pathophysiological and therapeutic mechanisms of coronary and cardiac diseases. Future studies may specifically focus on coronary large artery disease, coronary microvascular disease or heart failure and on the severity of these conditions.

In conclusion, the results demonstrate significant differences of the concentrations of all biomarkers investigated between the pericardial fluid and plasma, and significant different interplays between adipocytokines and inflammatory signals within each fluid compartment, indicating that the pericardial cavity is a distinct micro-environment of the heart in which leptin, lipocalin-2 and especially FABP4 seem to be derived from the heart.
